# Layer-by-Layer Nano-assembly: A Powerful Tool for Optical Fiber Sensing Applications

**DOI:** 10.3390/s19030683

**Published:** 2019-02-07

**Authors:** Pedro J. Rivero, Javier Goicoechea, Francisco J. Arregui

**Affiliations:** 1 Materials Engineering Laboratory, Department of Engineering, Public University of Navarre, Campus Arrosadía S/N, 31006 Pamplona, Spain; 2 Institute for Advanced Materials (INAMAT), Public University of Navarre, Campus Arrosadía S/N, 31006 Pamplona, Spain; 3 Nanostructured Optical Devices Laboratory, Department of Electric, Electronic and Communication Engineering, Public University of Navarre, Campus Arrosadía S/N, 31006 Pamplona, Spain; javier.goico@unavarra.es (J.G.); parregui@unavarra.es (F.J.A.); 4 Institute of Smart Cities (ISC), Public University of Navarre, Campus Arrosadía S/N, 31006 Pamplona, Spain

**Keywords:** optical fiber sensor, layer-by-layer, self-assembly, chemical sensor, biological sensor

## Abstract

The ability to tune the composition of nanostructured thin films is a hot topic for the design of functional coatings with advanced properties for sensing applications. The control of the structure at the nanoscale level enables an improvement of intrinsic properties (optical, chemical or physical) in comparison with the traditional bulk materials. In this sense, among all the known nanofabrication techniques, the layer-by-layer (LbL) nano-assembly method is a flexible, easily-scalable and versatile approach which makes possible precise control of the coating thickness, composition and structure. The development of sensitive nanocoatings has shown an exceptional growth in optical fiber sensing applications due to their self-assembling ability with oppositely charged components in order to obtain a multilayer structure. This nanoassembly technique is a powerful tool for the incorporation of a wide variety of species (polyelectrolytes, metal/metal oxide nanoparticles, hybrid particles, luminescent materials, dyes or biomolecules) in the resultant multilayer structure for the design of high-performance optical fiber sensors. In this work we present a review of applications related to optical fiber sensors based on advanced LbL coatings in two related research areas of great interest for the scientific community, namely chemical sensing (pH, gases and volatile organic compounds detection) as well as biological/biochemical sensing (proteins, immunoglobulins, antibodies or DNA detection).

## 1. Introduction

**Figure 1:**
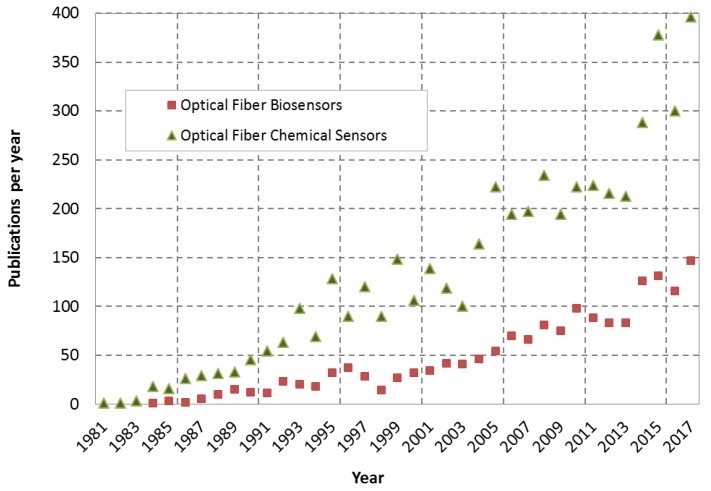
Evolution of the number of publications per year in the optical fiber biosensors and optical fiber chemical sensors research fields (source: Scopus).

Although the first experiments demonstrating the guiding of light by refraction took place in the XIXth century, the very first optical fibers were reported in the decade of the 1960s [1]. The initial research was focused almost exclusively on the study of optical fibers as waveguides to transmit data over long distances. It was in the decade of the 1970s when researchers started to look to the optical fiber field to create new sensor devices. The very first applications were related to the variations of the guided light (intensity, phase, polarization, etc.) due to the alteration of the waveguide properties of the optical fiber when it was submitted to different external physical changes (pressure, strain, temperature, etc). Consequently in this decade approaches of the first optical fiber gyroscopes [2], temperature sensors [3], or optical fiber hydrophones [4] were reported. Later, as optical fiber devices become more sophisticated (interferometers [5,6], gratings [7,8,9], special fibers [10,11], etc.), researchers started to use other approaches to create new optical fiber sensors combining the new optical fiber structures with other coating materials, functional layers, etc. With the combination of optical structures and new sensitive materials, new applications were reported such as chemical and biochemical optical fiber sensors. Since 1980 the number of research works on chemical and biological optical fiber sensors published in scientific journals has been growing consistently, as illustrated in Figure 1.

The irruption of nanotechnology in the decade of the 1990s was, without any doubt, a remarkable milestone in the history of optical fiber sensor research. The appearance of new materials and fabrication techniques that controlled the structure of the matter at the nanoscale level made possible the discovery of new materials with unique properties [12,13]. In this sense the use of nanoparticles, nanofibers, and ultra-thin films combined with the previous optical approaches yielded new sensing techniques [14,15,16] and new sensors with enhanced properties [17]. These technological advances in the development of smart materials have contributed to increase the applications of optical fiber sensors.

In this sense, in order to create new sensitive coatings for optical fiber sensor applications, the layer-by-layer (LbL) technique allows the design and fabrication of ultra-thin films of an enormous variety that can embed nanostructured materials. LbL can create nanofilms starting from a huge variety of materials such as polyelectrolytes, nanoclays, metallic or ceramic nanoparticles, carbon nanotubes, semiconductor quantum dots, chromophores, fluorophores, etc. This gives the opportunity of creating completely new composite nano-assemblies with adjustable properties that make possible the creation of an enormous variety of sensitive coatings. It has been also demonstrated that the LbL nanocoating properties can be adjusted, just by controlling the experimental conditions such as temperature, concentration, ionic strength, bifunctional molecules etc. Such parameters have a dramatic impact on the characteristics of the resultant LbL nano-assemblies (for example thickness and roughness), which are a critical aspect for the sensitivity and response times of the final sensor devices. Further details of the LbL technique and its applications will be commented in the following sections, and can be also be found in [18].

This ability to build highly controllable thin films with customizable composition, regardless of the size or shape of the substrate and using water as the main solvent of the process makes this technique especially attractive for the research of new optical fiber sensors. 

Given the importance of this topic, in this manuscript an up-to-date review of the main contributions that involve LbL optical fiber sensors is presented. This work has been structured in three different parts; a first one in which the main optical fiber sensor configurations are briefly described, and the two following sections that contain a comparative review on the most relevant works in LbL optical fiber chemical sensors, and optical fiber biological sensors, respectively.

### Optical Fiber Configurations

In this section a brief description of the main optical fiber sensor (OFS) schemes used with LbL can be found. This will help readers to understand the approaches discussed in Section 3.1 and Section 3.2 where chemical and biological sensor applications are summarized. In these applications LbL coatings are used to create optical fiber sensors where light is forced to interact with the sensitive coatings, inducing measurable changes in the guided light. Since standard optical fibers are specially designed for long-distance high speed data transmission, they are usually very robust waveguides almost totally immune to external interactions. That is the reason why many optical fiber sensor approaches use modified optical fibers were light interacts with an external sensitive coating.

**Figure 2:**
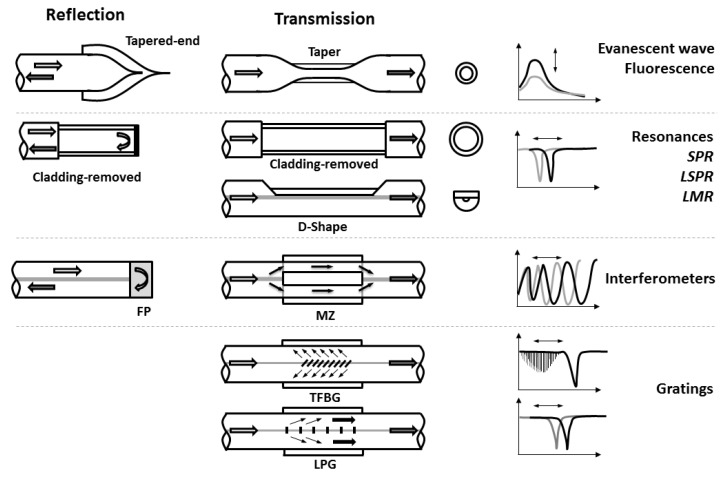
Schematic diagram that shows the main optical configurations that will be found in the applications sections. The light arrow indicates the light coming from the source, and the shadowed arrow indicates the light that goes to the detector. SPR stands for Surface-Plasmon Resonance, LSPR for Localized Surface-Plasmon Resonance, LMR for Lossy-Mode Resonance, FP for Fabry-Perot, MZ for Mach–Zehnder, TFBG for Tilted Fiber Bragg Grating and LPG for Long Period Grating.

Several optical fiber sensor configurations can be made attending to different aspects such as the position of the light source and the detector (transmission vs. reflection), or the nature of the sensing mechanism such as evanescent field, gratings, interferometers, just to mention a few. The information shown in this section is not an exhaustive classification attending to all possible attributes, rather than a brief summary that will be helpful in the following sections devoted to the different LbL optical fiber applications. These configurations are schematically shown in Figure 2, and it will useful as a guide for the following sections and tables, where the most relevant applications on optical fiber chemical and biochemical sensors based on LbL films are reviewed. In the following paragraphs, there is a brief description of the sensing principle for each main optical configuration mentioned.

The first type is the intensity-based optical fiber sensors. This kind of structure uses for example a coating that modifies the evanescent field losses of the fiber and most of the times uses U-bent fibers, tapered fibers, or cladding removed optical fibers in order to increase the magnitude of the evanescent field losses. These approaches are also used in fluorescence-based optical fiber sensors, in order to collect the light emitted from the fluorophore (or fluorescent nanocrystals) embedded into the LbL coating. In both approaches, the sensor information is coded in the intensity of the light signal.

Similar structures, most of the times in their transmission arrangements, are used to create sensors based on optical resonances, such as Surface Plasmon Resonances (SPR), Localized Surface Plasmon Resonance in nanoparticles (LSPR) and Lossy-Mode Resonances (LMR). Those structures have the advantage of their wavelength-based nature, which is more robust than the intensity-based optical fiber sensors (prone to noise, light-source instability dependence, etc.). The sensing signal here is coded into the wavelength-shift of the resonance bands, although some intensity-based approaches can be also found.

Other approaches use the separation of light into several optical paths, creating an interference pattern when the different paths interact. In reflection arrangements it is very common to find the Fabry-Perot (FP) one, but it is also possible to find an in-line version of the FP interferometer using a short segment of multimode fiber (cavity) inserted between two single mode fibers (SMS structure). A different interferometer can be also found with a Mach–Zehnder (MZ) configuration where the injected light is forced to be guided through the cladding of a short segment of fiber and then refocused into the core again. The change in the optical paths for example due to an external effective index variation through the cavity modify the interference patterns, which give wavelength-dependent signals. 

Finally it is possible to create sensors with optical fiber gratings. The first Fiber Bragg Grating (FBG) was demonstrated by Hill in 1978 [19]. In FBGs the light is confined in the core of the fiber, consequently their use is more restrictive for sensing applications, being commonly used as temperature and strain intrinsic optical fiber sensors. Nevertheless Tilted Fiber Bragg-Gratings (TFBGs) and Long-Period Gratings (LPGs) create different kind of cladding propagating modes that are able to interact with external sensitive coatings. In such devices small variations on the external effective refractive index, for example due to the alteration of a sensitive coating, can alter the resonance condition of the grating causing a measurable wavelength shift in the optical spectrum of the transmitted light.

## 2. Fundamentals of the Layer-by-Layer Technique

The LbL assembly is a nanofabrication technique based on the alternate immersion of the substrate into aqueous polyelectrolyte solutions with opposite electric charge, being the electrostatic interaction the main force to adsorb onto a surface for obtaining a polyelectrolyte multilayer (PEM) structure [20]. This technique is considered as a “wet process” because the materials (polyelectrolytes) used for the fabrication of a multilayer assembly are found in the form of aqueous solutions [21,22]. This nano-assembly technique offers several advantages in comparison with conventional methods for the fabrication of optical thin films. First of all, LbL process is a very versatile technique because it can be implemented over a wide range of substrates (glass, ceramics, wood, metals, plastics, semiconductors) [23] of almost any shape or size (i.e., not only limited to planar substrates). In addition, one great advantage is that the process is extremely cost effective and the resultant LbL films show a high stability because their structural ordering does not decay over long-storage periods [24]. Furthermore, this LbL technique can be performed at room temperature and pressure, and a high degree of control over the thickness can be precisely obtained with even 1 nm resolution, producing well-organized structures with a high uniformity. Other remarkable aspect is that this repetitive and highly reproducible technique is easily scaled up for high throughput fabrication without using sophisticated instrumentation or equipment. Finally, this technique is considered as an ecofriendly environmental alternative because it is based on a water soluble process without any volatile organic compounds. 

### 2.1. Polyelectrolytes

**Figure 3:**
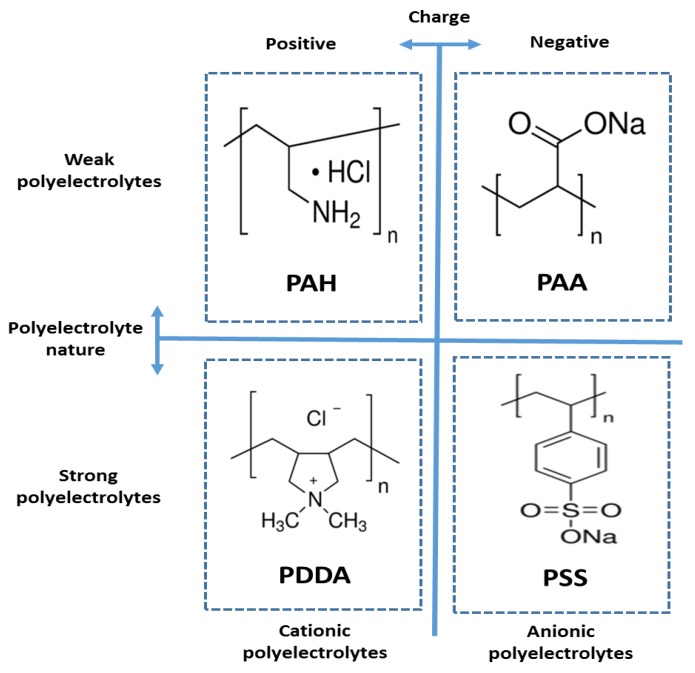
Schematic representation of the constitutional repeat unit for the most representative cationic polyelectrolytes (PAH, PDDA) and anionic polyelectrolytes (PAA, PSS) used in the LbL deposition technique, showing their corresponding behavior as weak or strong polyelectrolytes, respectively.

Polyelectrolytes are polymers with ionizable functional groups that form charged polyions (with overall positive or negative charge), thus constituting either polycations or polyanions, respectively. According to this, a substance with positively charged functional groups (mostly quaternary ammonium or amino groups) is used as a polycation, being the most significant examples poly(allylamine hydrochloride) (PAH), polydiallyldimethylammonium chloride (PDDA) and poly(ethyleneimine) (PEI); whereas a substance with negatively charged groups (mostly sulfonic acids or carboxylic acids) are the most common functional groups used in polyanions, being the most common examples poly(sodium 4-styrene sulfonate) (PSS) and poly(acrylic acid sodium salt) (PAA). In addition, an important aspect to remark related to these polyelectrolytes is that these charged polymers can be classified as strong or weak polyelectrolytes, respectively. According to this classification, polyelectrolytes that maintain a high and constant ionization ratio over a broad range of pH conditions are termed as “strong polyelectrolytes” (being the most representative examples PDDA or PSS), whereas polyelectrolytes that exhibit pH-dependent ionization degrees are called “weak polyelectrolytes” (being the most known examples PAH or PAA), respectively. A summary of the chemical structure (constitutional repetitive unit) of the most common polyelectrolytes used for the fabrication of a multilayer structure by the LbL technique can be appreciated in Figure 3. Other important aspect to consider is that a long list of other different positive or negative polyelectrolytes which can be also employed for the buildup of thin films can be found in the literature, being some representative examples poly(vinyl pyridine) (PVP), poly(vinyl sulfate (PVS), poly((2-(methacryloyloxy)ethyl) trimethylammonium chloride) (PCM), poly- (phenylene vinylene) (PPV), poly(methacrylic acid) (PMA), poly(sodium phosphate) (PSP), sulfonated polyaniline (SPAN), or poly(4-vinylbenzyltrimethylammonium chloride) (PVBTA), among others.

The overall charge of the weak polyelectrolyte solutions can be tuned as a function of the pH and the ionic strength of the dipping solutions as far as these two parameters directly influence in the conformation and mutual interactions of the polymeric chains [25,26], offering a chance to adjust the thickness, the roughness or the porosity of the LbL coating. Other works are focused on the design of hydrogen-bonded (HB) LbL films thanks to the combination of a weak polyelectrolyte (mostly of polycarboxylic acid nature) with neutral polymers such as poly(vinylpirrolidone) (PVPON), poly(vinyl alcohol) (PVA), poly(ethylene oxide) (PEO) or poly(acrylamide) (PAAM) [27,28], and as a result, the properties of these hydrogen bonded materials can respond strongly to environmental stimuli [29,30]. 

### 2.2. Implementation of the Layer-by-Layer Technique in Optical Fiber Sensors

The whole fabrication process involves sequential and ordered steps by using an immersive technology, although this fabrication process can be also extrapolated to other alternative such as spray assembly by aerosolizing polymer solutions in a sequential order onto the substrates [31,32]. In this review work, due to the small dimensions of the reference substrate (in our case optical fiber) [33], the immersion technology for the fabrication of LbL coatings has been selected because this approach makes possible a better and easier manipulation of the optical fiber, as it will commented in the following sections.

In order to perform the experimental procedure, first of all, the substrate (optical fiber) is cleaned and treated to create an electrically charged surface, for instance a negative charged surface. Then, the substrate is exposed to a solution of a polycation for a short period of time (minutes) and as a result, a monolayer of polycation is adsorbed by electrostatic attraction on the surface, reversing the original electrical charge. After that, the substrate is immersed into a solution of polyanion and, by adsorption of a monolayer of polyanions, and as a result, the original negative charged surface is restored. In this way, the substrate is alternately dipped into solutions of cationic and anionic polymers in order to create a multilayer thin film. The pair of one cationic monolayer and one anionic monolayer is called bilayer. Additionally, after each immersion in the dipping polyelectrolyte solution, the substrate is rinsed with ultrapure water in order to remove the excess of non-adsorbed material onto the substrate. This process can be repeated up to the desired thickness as a function of number of bilayers during the fabrication process. Other aspect to remark is that a number of parameters have to be perfectly controlled in order to achieve repeatable thin films, such as concentration of the dipping polyelectrolytes solutions, pH, curing temperature, immersion time, and ionic strength, among others [34,35,36,37]. 

**Figure 4:**
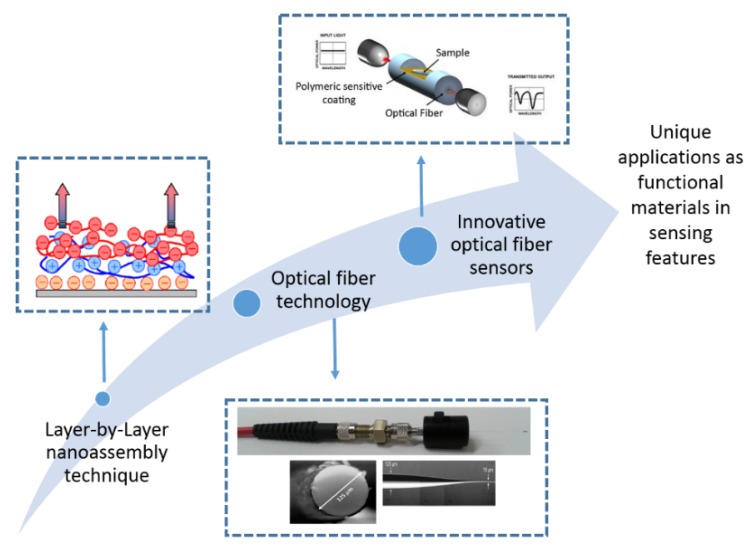
Potential applications of innovative optical fiber sensors (industry, medicine, biology, agriculture) as a function of the implementation of nanostructured thin films by LbL nanoassembly onto optical fiber.

The great versatility combined with the simplicity and easily scalability of the LbL technique makes it possible that this deposition technique to be used as a suitable method for fabricating optical fiber sensors based on the development of nanostructured thin films [38,39,40]. The control of the structure of the coating at the nanoscale level allows the ability to tailor at the molecular level of the composition and structure of the sensitive coatings. The implementation of nanoscaled materials shows a special attraction because there are new phenomena observed when the matter is downscaled to this scale [41,42]. Consequently, research in nanostructured sensitive LbL coatings is a hot and challenging topic in the scientific community because these LbL sensitive nanocoatings can modify their optical properties as a function of the target parameter of analysis with an important enhancement in the response time, robustness, selectivity and sensitivity [43,44]. Such improved LbL-based sensors also show the classical advantages of the optical fiber sensors, such as immunity to electromagnetic interferences, remote sensing, easy multiplexing, portability, light weight, small size, long-distance transmission, resistant to corrosive environments, etc. [45,46]. As a consequence, the number of research papers related to optical fiber devices based on LbL coatings has been recently increased and different type of optical fiber sensors have been reported for the detection of a wide variety of biological, chemical or physical parameters by using this specific nanofabrication technique [47,48,49,50]. In Figure 4, a schematic summary of the main objectives of this work, which is based on the combination of both LbL nanoassembly technique and optical fiber technology for the development of novel optical fiber sensors, is presented.

### 2.3. Examples of Advanced Layer-by-Layer Coatings 

**Figure 5:**
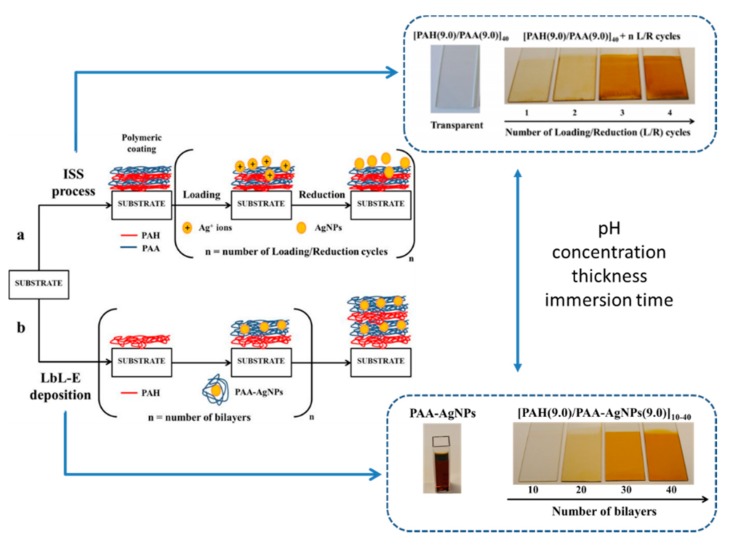
Schematic representation of two different chemical synthetic routes for the incorporation of silver nanoparticles (AgNPs) into LbL films by using the in situ synthesis (ISS) process (**a**) and LbL embedding (LbL-E) deposition technique with the corresponding aspect of the resultant coatings as a function of several experimental parameters (pH, concentration, thickness, immersion time). Reprinted from [71] with permission.

An important consideration related to the LbL technique is that it can be implemented on a wide variety of different materials (not only polyelectrolytes), making possible the fabrication of advanced LbL coatings. According to this, a wide number of scientific research papers based on the incorporation of metal nanoparticles [51,52,53,54,55], carbon nanotubes [56,57], metallic oxide nanoparticles (silica, titania) [58,59,60,61,62], organic dyes [63], polypeptides [64], proteins [65,66], DNA [67], dendrimers [68], or quantum dots [69,70], among others, can be found in the literature. A representative example of this high versatility is shown in Figure 5 where two different chemical approaches denoted as the in situ synthesis (ISS) process and LbL embedding (LbL-E) deposition technique are used for the incorporation of metallic silver nanoparticles (AgNPs) into LbL films [71]. 

**Figure 6:**
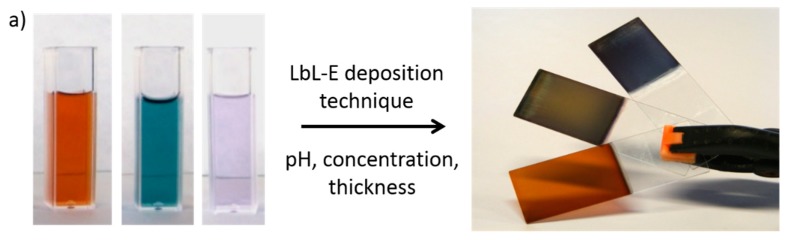
(**a**) Multicolored silver nanoparticles (PAA-AgNPs) (orange, green and violet) used for the LbL-E deposition technique and the final aspect of the multicolored LbL-E thin films [PAH/PAA-AgNPs]_80_ with orange, green and violet coloration, respectively; (**b**) Aspect of the PAA-AgNPs with a spherical shape (orange), hexagonal shape (green) and rod shape (violet). Reprinted from [73] with permission.

An important advantage related to the LbL-E deposition technique is that AgNPs with a specific shape can be previously synthesized [72], and then, these AgNPs can be successfully embedded into LbL thin films, thanks to a good control over experimental parameters, obtaining multicolor LbL coatings [73]. A representative example is observed in Figure 6 where three different multicolored silver nanoparticles (orange, green and violet coloration), denoted as PAA-AgNPs, have been firstly synthesized with a specific shape (spherical, hexagonal and rod), making possible the fabrication of multicolored LbL-E thin films with similar coloration as their respective AgNPs colloidal dispersions after 80 bilayers, denoted as [PAH/PAA-AgNPs]_80_.

**Figure 7:**
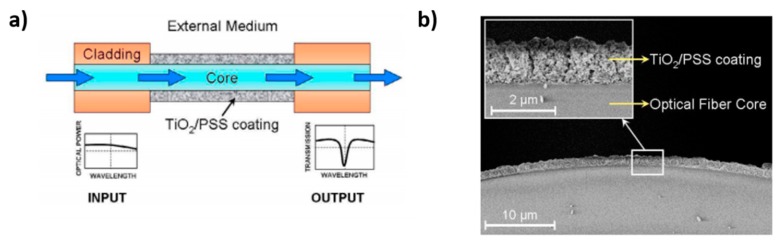
(**a**) Schematic representation of the optical fiber device; (**b**) SEM image and detail of the coated optical fiber core. Reprinted from [74] with permission.

A schematic representation of the fabrication of an advanced LbL coating onto an optical fiber core (reference substrate) for the design of an optical fiber refractometer can be observed in Figure 7 [74]. In this case, water solutions of TiO_2_ nanoparticles and poly(sodium 4-styrenesulfonate) (PSS) have been used as the cationic and anionic solutions, respectively. In this work, the LbL technique makes it possible to tune the sensitivity and the operation wavelength of the device, which was sensitive to changes in the refractive index (RI) of the surrounding medium, showing a sensitivity of 2228 nm/RIU. Other significant example can be found in [75] where it is presented the combination of a fluorescent pH indicator (8-hydroxypyrene-1,3,6-trisulfonic acid trisodium salt, HPTS) with an hydrophilic nanostructured coating composed of silica nanoparticles (denoted as S2 device), and as result, a considerable change in the topographic surface has been observed in comparison with a device with only HPTS coating (S1 device). 

**Figure 8:**
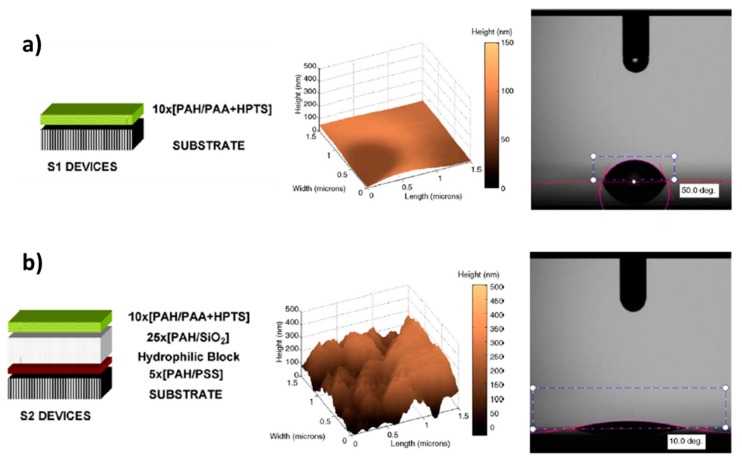
Schematic representation of the coating fabrication by using the LbL technique (left), AFM images of the corresponding coatings (middle) and contact angle measurements (right) for S1 device (**a**) and S2 device (**b**), respectively. Reprinted from [75] with permission.

In Figure 8, a schematic representation of the build-up for both devices (S1 and S2 devices) as well their corresponding AFM images and contact angle measurements is shown. The S2 device shows a higher average roughness than S1 devices (see AFM images), and the presence of the hydrophilic block (inherent to the silica nanoparticles) in S2 results in a lower water contact angle value (10°) which is directly associated to the higher average roughness in comparison with the contact angle value of S1 device (10°). Finally, these are some representative examples of the wide applicability related to the LbL nanoassembly, although other different nanomaterials (even of a biological nature) can be successfully fabricated as it will be presented in the following section.

## 3. Applications of the Optical Fiber Sensors Based on Layer-by-Layer Coatings

In this section, some relevant LbL optical fiber applications in fields as diverse as biology, chemistry, engineering, industry or medicine will be briefly analyzed. In a first subsection, optical fiber chemical sensors for pH detection are presented. In a second subsection, optical fiber sensors for gas and volatile organic compound (VOC) detection are presented. Finally, in the third subsection, optical fiber biosensors for the detection of biological and biochemical parameters are presented. 

### 3.1. Chemical Sensing Applications

#### 3.1.1. pH Sensing Applications

pH monitoring is an essential task in industrial processing (i.e., water quality, food production processes, oil or gas industry) as well as in biomedical research (blood tests, tissue metabolism, cancer diagnosis) in which a real-time measurement is needed and thus a quick, minimum invasive impact and high resolution sensor is necessary. 

**Figure 9:**
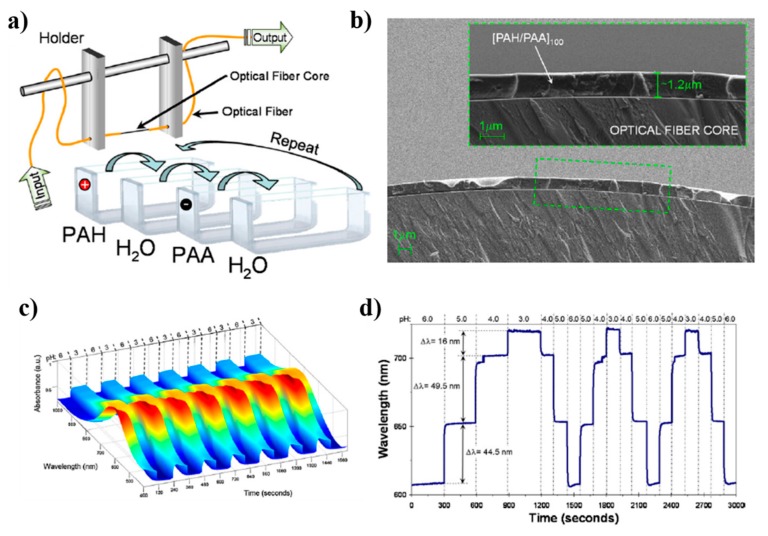
(**a**) Schematic representation of the fabrication procedure onto optical fiber core (sensitive region) by using weak polyelectrolytes such as PAH and PAA, respectively; (**b**) SEM image of the resultant LbL thin film fabricated onto the optical fiber core; (**c**) spectral response when the sensitive region is immersed alternatively into pH 6 and pH 3 solutions at different time intervals; (**d**) dynamic response as the pH of the surrounding medium is cycled from pH 6 to pH 3 every pH unit for different times. Reprinted from [81] with permission.

One of the most common polyelectrolytic pairs used for pH sensing is the (PAH/PAA) one due to its intrinsic swelling pH behavior of the resultant LbL films, although this swelling behavior is highly non-linear with the pH [25], and consequently the response curve of the sensor also shows that non-linearity (measured as absorbance changes) [76]. However, very recently, a new optical phenomenon, known as Lossy Mode Resonance (LMR), has attracted the attention of the scientific community within the last years due to its intrinsic versatility, high sensitivity and robustness [77,78]. This LMR phenomenon occurs when the real part of the thin-film permittivity that coats an optical waveguide is negative and higher in magnitude than both its own imaginary part and the real part of the permittivity of the material surrounding the thin-film [79]. According to this, several works have studied the importance of using this LMR absorption band as a sensing signal with a high wavelength dependence, being possible to tune the wavelength position of the LMR absorption peaks as a function of the resultant thickness of the LMR-supporting coating [80]. The first work based on the generation of LMRs by means of (PAH/PAA) polymeric pH sensitive coating can be found in [81], showing an average sensitivity of 0.027 pH units/nm with accuracy of ±0.001 pH units, good repeatability and fast response within the pH range from 3.0 to 6.0, as it can be appreciated in Figure 9. 

**Figure 10:**
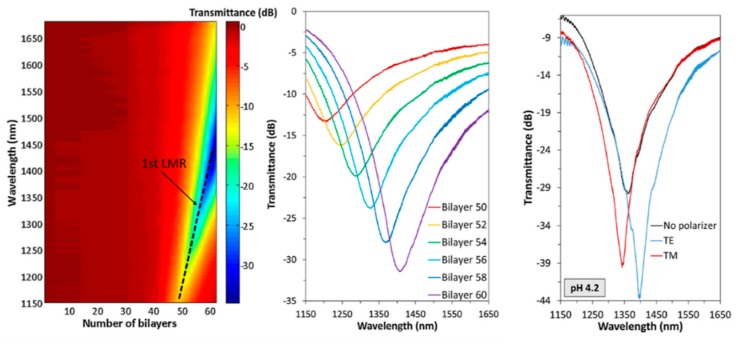
Evolution of the transmitted spectra during the deposition of PAH/PAA polymer chains at pH 4.0 and separation of the TM (red) and TE (blue) mode resonance components when the sensitive region is immersed in a solution at pH 4.2. Reprinted from [86] with permission.

**Figure 11:**
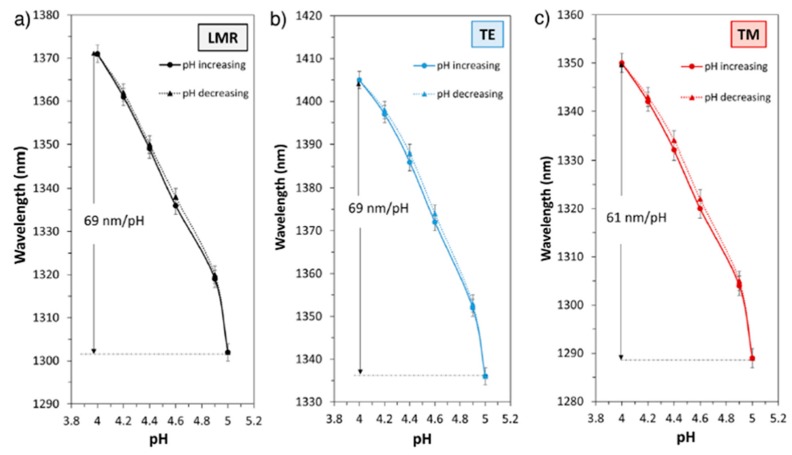
Response of the device B for LMR without polarization controller (**a**), LMRTE (**b**) and LMRTM (**c**) when the sensitive region is immersed to successive pH changes between pH 4.0 and 5.0 (variations of 0.2 pH units). Reprinted from [86] with permission.

Further studies have been focused on the use of this same pH sensitive coating of (PAH/PAA), although it has been implemented over other different optical fiber configurations [82,83,84,85,86,87]. A representative example can be found in [84] where a high sensitive optical fiber pH sensor based on the combination of both tapered single-mode optical fibers (T-SMF) and LMR phenomenon for pH detection is presented, reaching a wavelength shift of 200 nm within the pH range from 4.0 to 6.0, respectively. Other interesting works can be found in [85,86] where LMR supporting coatings (PAH/PAA) are deposited onto D-shaped optical fibers, showing an optical wavelength shift of the LMR when the pH is increased with 0.2 pH variations. Another advantage related to the LMR phenomenon is that it can be generated with both transverse electric (TE) and transverse magnetic (TM) polarization light and they can be tuned to different wavelengths of the spectrum [87]. A representative example related to both LMR_TM_ and LMR_TE_ can be found in [86] where the sensitivities of the LMR_TM_ and LMR_TE_ in two pH ranges (from 4.0 to 5.0 and from 7.0 to 8.0) have been measured by using two different devices fabricated by PAH/PAA polymer chains at pH 7.0 (device A) and at pH 4.0 (device B). The experimental results indicate that best sensitivity with a low hysteresis (0.04%) has been obtained for device B, being for LMR_TM_ (61 nm/pH) and LMR_TE_ (69 nm/pH) in the pH range from 4.0 to 5.0 in comparison with the device A with a sensitivity of 30 nm/pH (LMR_TM_) and 34 nm/pH (LMR_TE_) in the pH range from 7.0 to 8.0, respectively. In addition, the monitoring of both LMR_TM_ and LMR_TE_ resonances makes possible to perform dual measurements, which could be helpful to reduce noise or extend the dynamic range of the devices. In Figure 10, the evolution of the transmitted spectra during the fabrication process as well as the transmittance obtained with the device B (black line) with its corresponding separation of LMR_TM_ resonance (red line) and LMR_TE_ resonance (blue line) when the sensitive region is immersed in a pH solution of pH 4.2 is presented. In Figure 11, the resonance wavelength shift of the device B when the sensitive region is immersed in different pH solutions for LMR (without polarization controller), LMR_TE_ and LMR_TM_, respectively, is presented.

**Figure 12:**
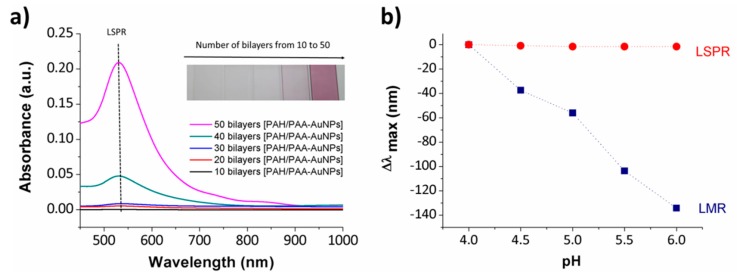
(**a**) UV-Vis spectra of the nanostructured coating obtained by LbL Embedding (LbL-E) deposition technique for different number of bilayers and a photograph of the resultant nanocoatings; (**b**) maxima wavelength variation of both LSPR and LMR absorption bands for pH changes from pH 4.0 to 6.0, respectively. Reprinted from [88] with permission.

Other interesting works which employ the LbL technique for the development of hybrid nanostructured coatings based on both inorganic nanoparticles (gold nanoparticles, AuNPs) and organic polymeric structure (polyelectrolytes) onto the optical fiber core for pH sensing can be found in [88,89]. Due to the special optical properties inherent to the metallic nanoparticles, a Localized Surface Plasmon Resonance (LSPR) band is appreciated at a specific wavelength position (around 530 nm) which is indicative that spherical PAA-AuNPs have been successfully incorporated in the multilayer structure [90]. In addition, when the resultant thickness coating is increased up to 20 bilayers the LMR phenomenon can be also observed during the fabrication process. The experimental results indicate that the LMR sensitivity is very high (67.35 nm/pH unit) in comparison with the low sensitivity of the LSPR band (0.75 nm/pH unit). This great difference in the resultant sensitivity makes possible to consider a dual reference sensor, in which the LMR band has a significant sensitivity to pH changes, whereas the LSPR band can be used as a wavelength reference signal due to its small sensitivity, as it can be clearly observed in Figure 12.

Another of the most common strategies for the design of effective optical fiber pH sensors is based on the use of specific pH indicators such as Prussian Blue (PB) [91], Neutral Red (NR) [76,92,93,94] or Brilliant Yellow (BY) [95,96] as well as fluorescent pH indicator such as HPTS [75,97], among others. In several works, the specific pH indicator can be perfectly dissolved in one of the polyelectrolytes, as it can be observed in [91,92,97]. A representative example with PB dissolved in a positive polyelectrolyte such as PAH is presented in [91] where linear pH sensors in the pH range from 4 to 7 have been obtained, showing a good repeatability. In others works [92,93], it has been demonstrated that NR can be also dissolved in the polycation PAH, forming the specific sensitive structure of (PAH+NR/PAA). By using this specific sensitive coating, a wide pH sensing range (from pH 5 to 10) with a fast response is presented in [93].

**Figure 13:**
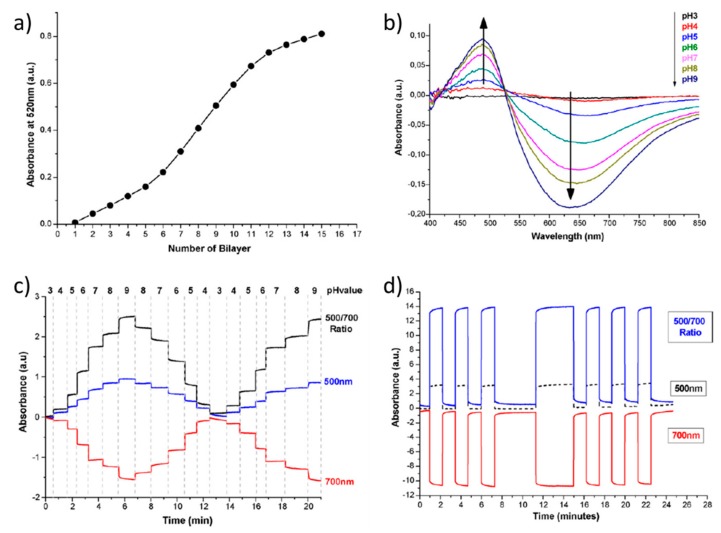
(**a**) absorbance at 520 nm when the number of bilayers is increased during the fabrication process in the (NR/PAA)_15_ device; (**b**) absorbance spectra of the (NR/PAA)_15_ devices when the sensor is immersed into different pH buffer solutions; (**c**) dynamic response of the (NR/PAA)_15_ device when is immersed into buffer solutions from pH 3 to pH 9; (**d**) repetitive response of the (NR/PAA)_15_ device when is immersed alternately into pH 3 and pH 8 buffer solutions. Reprinted from [76] with permission.

However, an important advantage of the use of specific pH indicators such as NR or BY is that both can be employed as either polycations or polyanions as a function of their intrinsic chemical structure. In this sense, NR is a molecule that shows a positive charge and its color changes from red at pH 6.8 to yellow at pH 8.0, showing an absorption peak located at 522 nm in the UV-Vis spectroscopy. A first work by using NR as a polycation and PAA as a polyanion can be found in [76], being the corresponding multilayer structure (NR/PAA)_15_, showing faster response times, high repeatability and low hysteresis, as it can be appreciated in Figure 13. In Figure 13a, the increase of the absorbance at 520 nm (related to the NR molecule) when the number of bilayers is increased is plotted, showing at the sensor tip a reddish color visible to the naked eyes. In Figure 13b, it can be observed that the absorbance spectra showed a characteristic behavior when the device (NR/PAA)_15_ was immersed in the buffer solution because a peak around 480 nm was gradually grown when the pH was increased, whereas a valley near 650 nm showed a decrease in absorbance as a function of higher pH values. This fact can be used for improving the sensitivity of the sensor by using, instead of the absorbance at only one wavelength, the ratiometric measurement between the peak (480 nm) and the valley (650 nm) of the absorbance spectrum. In Figure 13c, the dynamic response of the sensor is shown, where an optimized signal can be observed which is a ratio between the reflected optical power at 500 nm respect to the one at 700 nm, giving as a result a very high quality response, with a dynamic range of about 2.5 dB and almost negligible drift which suggests a high robustness of the LbL coatings. In addition, the response time resulted to be very fast, almost instantaneous (less than 1 s for all pH values). Finally, a highly repetitive behavior (see Figure 13d) is observed by using a same pH step routine (pH 3 and 8, respectively), and the sensor reacted with very short response times (less than 1 s). In other work [94], by using this same multilayer structure of (NR/PAA), two different approaches such as the addition of salt into the polyanion solution and the use of different drying methods are evaluated with the aim to improve the optical properties of the pH-sensitive coating. According to this, the addition of salt which provokes rougher and more porous coatings has produced an enhancement in the sensitivity of the probe from 65 nm/pH unit (no salt) up to 105 nm/pH unit (0.15 M) with a shift in the sensitivity range, whereas drying with nitrogen presents the largest influence on both peak and wavelength because there is a better control over the swelling/deswelling behavior. 

Another interesting pH indicator is Brilliant Yellow, which is of great interest because according to its chemical structure, this molecule is negatively charged due to the presence of two sulfonate groups, making possible its use as an effective polyanion during the fabrication process of a multilayer structure. In addition, its color changes from yellow at pH 6.4 to red-orange at pH 8.0, showing an absorption peak located at 497 nm in the UV-Vis spectroscopy. Raoufi et al. have reported in [95] a wavelength-dependent pH optical sensor which is fabricated by combining BY and PAH as a cross-linker at the end of a bare silica core. The main conclusions derived from this work is that a thickness coating of six bilayers, denoted as (PAH/BY)_6_, has shown the best sensitivity for a pH range from 6.80 to 9.00 with an average wavelength shift of 4.65 nm per 0.2 pH units. In a later work [96], by using the same reagents for the fabrication of the LbL coatings, a number of key parameters such as the number of bilayers, the concentration of the BY solution, the shape of the fiber (U-bend or straight) and the variation of the core diameter have been evaluated in order to optimize the design and performance of the resultant pH optical fiber sensors. The main conclusion derived from the experimental results is that the sensitivity can be enhanced by curving the fiber to a U-bend shape with a smaller radius in comparison with straight shape, by using a lower concentration of BY solution (0.25 mM instead of 0.5 mM) and by increasing the number of bilayers up to a total thickness of six bilayers, respectively. The best sensitivity was obtained for a pH range from 7.00 to 9.00 with an average wavelength shift of 5.45 nm per 0.2 pH units. It is important to remark that in [95,96], the resultant LbL coatings have been cured at 120 °C in order to improve the stability of the film and to avoid progressive degradation of the indicator. 

Other works are devoted to the immobilization of a fluorescent pH sensitive dye such as 8-hydroxypyrene-1,3,6-trisulfonic acid trisodium salt (HPTS) for the development of pH optical fiber sensors. In addition, the presence of silica nanoparticles (SiO_2_NPs) into LbL coatings has produced an important enhancement in the response time of the resultant optical fiber pH sensor due to the inherent hydrophilic behavior of this type of metal oxide nanoparticles. This aspect has been observed in [75] where two different sensors denoted as S1 (only with HPTS fluorescent pH indicator embedded in a LbL polymeric matrix) and as S2 (combination of the same HPTS-polymeric matrix and SiO_2_NPs) have been tested. As result, the rise time response to pH changes from 3 to 7 has been shortened by a factor of five, from 15 min (S1) to 3 min (S2) and the fall time response to pH changes from 7 to 3 has been also shortened by a factor of three, from 3 min (S1) to 1 min (S3). Another interesting work using this same fluorescent pH indicator (HPTS) is presented in [97] where the main objective was to minimize the photobleaching of the sensitive coating by using both a thermal curing step and the addition of an antifading agent such as 1,4-diazabicyclo [2.2.2]octane. 

It is important to remark that other different pairs of polyelectrolytes (not only PAH and PAA) can be also used for sensing purposes [98,99,100,101]. According to this, a novel fiber optic pH sensor composed of a multilayer of poly(diallyldimethylammonium chloride) (PDDA) and poly(acrylic acid) (PAA) on a titled fiber Bragg grating (TFBG) is presented in [98], showing a sensitivity of 117 arbitrary units (a.u.)/pH unit in the range of pH from 4.66 to 6.02 with a dynamic response of 10 s (rise time) and 18 s (fall time), respectively. An interesting work using the same polyelectrolytes (PDDA and PAA) can be also found in [99], although therein a strategy for preparing a novel fast response fiber-optic sensor by constructing a nanoporous multilayer film onto the thin-core fiber modal interferometer (TCFMI) is presented The resultant coating is prepared by depositing a polycation blend of poly(4-vinylpyridiniomethanecarboxylate) (PVPMC) and PDDA with PAA as a polyanion. After that, the resultant nanoporous films were obtained by sacrificing a PVPMC/PAA template from the PVPMC+PDDA/PAA multilayer film as a function of a physiological condition (i.e., 0.15 M NaCl solution). This strategy for obtaining nanoporous films makes possible the design of fiber-optic pH sensors which can reduce greatly the response time (rise time of 20 s and fall time of 15 s) in comparison with fiber-optic pH sensors composed of dense non-porous films with a rise time of 240 s and a fall time of 160 s, respectively. In work [100] a new fiber-optic pH sensor composed of positively charged PDDA and negatively charged polyelectrolyte complex (PEC^−^) nanoparticles, made of sodium carboxymethyl cellulose and PDDA, is presented on the surface of TCFMI. The presence of these PEC^-^ nanoparticles into the sensitive coating produces an enhancement in the root mean square (RMS) surface roughness, making possible a faster response time in comparison with only polyelectrolyte coating. Finally, other interesting work is presented in [101], where two biocompatible polyelectrolytes such as sodium alginate (SA) and polyethylenimine (PEI) have been used for the fabrication of the sensitive coating onto the thin-core fiber modal interferometer (TCFMI). The experimental results indicate that the optical fiber pH sensor shows a linear, fast, reversible and long-term stability in a large pH range (from pH 2 to 11) which makes the biosensor very promising for biomedical and clinical applications. 

In order to have a better understanding of the different optical fiber pH sensors analyzed in this section, a summary of the different sensitive coatings employed as well as the optical structure and the sensing mechanism for a studied pH range is shown in Table 1. 

#### 3.1.2. Gas and Volatile Organic Compound (VOC) Sensing Applications

Volatile organic compounds (VOCs) are organic chemical compounds whose composition makes it possible for them to evaporate under normal indoor atmospheric conditions of temperature and pressure. These substances are easily inhaled, explosive, and in many cases, toxic (depending on the concentration and exposure time) [102]. In fact, most of the chemical solvents used to synthesize products of our daily life (i.e., lacquers, paints, cleaning products, food processing, cosmetics, and pharmaceuticals, among others) are also volatile. The high toxicity and their corresponding environmental impact have forced governments to legislate their emissions and for this reason, it is necessary to control these vapor concentrations continuously in places where they are handled because a long exposure even to low concentrations of several VOCs can produce health disorders [103,104]. 

On the contrary, a gas like oxygen plays a key role in human live, as well as in many biological processes, chemical and biochemical reactions [105]. Thus, its monitoring is of great interest in different areas, such as biology, medicine, agriculture or food industry among others [106,107]. One of the most employed strategies for oxygen detection is the design of luminescence optical fiber sensors where a specific oxygen sensitive fluorophore (mostly of a porphyrin nature) is embedded into the polymeric LbL matrix [108,109,110,111,112]. One of the most used luminescent materials for oxygen detection is [Ru(bpy)_3_]^2+^ which emits phosphorescence at the wavelength of around 620 nm by means of irradiating blue light at the wavelength of around 450 nm. An interesting work by using this specific luminescent material can be found in [108] where an optical fiber oxygen sensor by using stacked porous composite membranes is presented as a function of the resultant thickness (5, 50 and 125-layers) obtained by LbL technique. In this work, the porous composite membrane has been fabricated by a mixture of porous glass beads and poly(allylamine) as polycation and poly(acrylic acid) as a polyanion. The experimental results indicate that 5-layer and 50-layer sensors showed the best sensitivity and reproducibility, showing a decrease of the optical intensity at the wavelength of 625 nm when the oxygen concentration is increased. 

**Figure 14:**
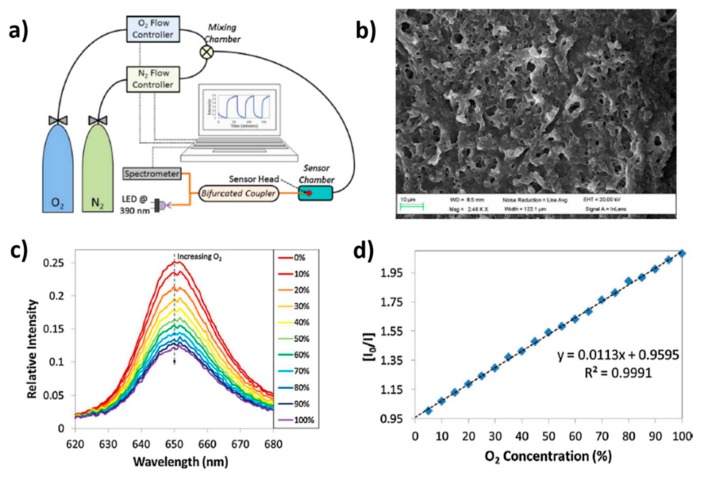
(**a**) Experimental set up used to characterize the sensor. The flow controllers are electronically tuned by software; (**b**) SEM image of the resultant coating obtained from an optical fiber once the construction process has been performed; (**c**) Luminescence signal from the sensor when it is exposed to different oxygen concentrations; (**d**) Stern–Volmer plot obtained from the sensor for increasing oxygen 5% steps. Reprinted from [110] with permission.

This same behavior related to optical emission band of the luminescent material as a function of gradual increase of the oxygen concentration is observed in [109,110,111,112]. In these works, the selected luminescent complex is platinum tetrakispentrafluorophenyporphine (known as PtTFPP) because it shows excellent features such as long lifetime emission, photo stability and significant Stokes shift (around 250 nm). In Figure 14, the experimental setup used for oxygen detection (Figure 14a) as well as the morphology of the coating by using a SEM (Figure 14b) can be appreciated. In addition, the emission peak is centered at 650 nm, showing a reduction in intensity when the oxygen concentration is increased (Figure 14c), what is expected due to the quenching effect of oxygen, whereas a linear relationship between the intensity of the emission and the resultant gas concentration (Figure 14d) can be clearly observed [110]. In a later work [111], it is presented a comparative study of different polymeric matrices embedding the same oxygen-sensitive fluorophore (PtTFPP), changing the resultant polycation (PAH, PDDA and PEI, respectively). The results indicated that a remarkable difference on the kinetics, resolution and sensitivity of the sensors have been obtained as a function of the polymeric matrix used and the rougher and thicker PAH coatings were the ones with shorter response times, better resolution and sensitivity. Another novel work can be found in [112], where an enhancement of luminescence-based optical fiber oxygen sensor has been obtained by an adequate tuning of the distance between the fluorophore layers just by introducing additional polymeric LbL layers that act as spacers. 

**Figure 15:**
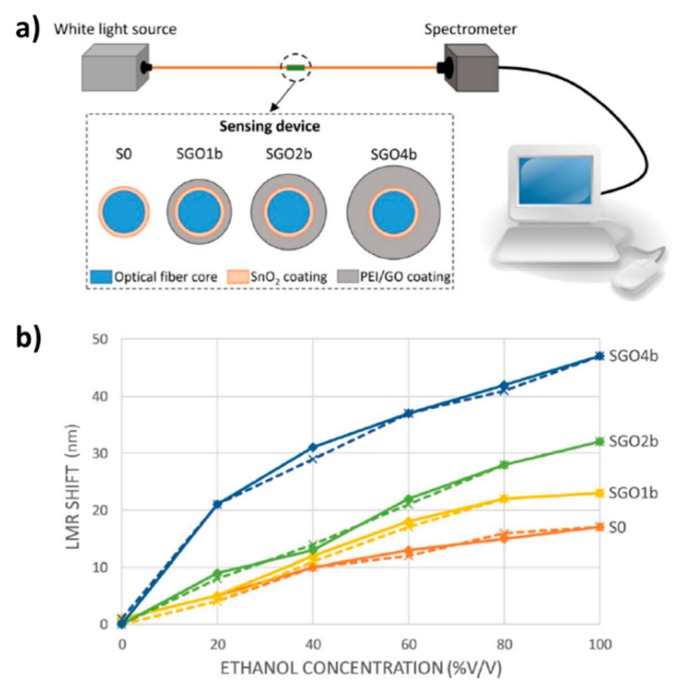
(**a**) Optical fiber experimental setup and sensors schematic structure; (**b**) Shift of LMR absorption peak generated by the four different sensors when they are immersed in ethanol solutions with different concentrations from 0 to 100% *v/v* (straight lines) and from 100 to 0% *v/v* (dashed lines). Reprinted from [116] with permission.

Ethanol is other chemical compound commonly used in many fields such as medicine, food processing, the beverage industry or even as an increasingly important biofuel [113,114]. Its high flammability and volatility make optical fiber sensors ideal candidates for the safe and accurate measurement of this substance [115,116]. In these works a novel LMR optical fiber ethanol sensor based on a first step of sputtered SnO_2_ coating, and second step, a multilayer structure of polyethyleneimine (PEI) and graphene oxide (GO) is coated onto the SnO_2_ layer is presented. A comparative study about the resultant shift of the LMR absorption band for four different sensors denoted as SO (17 nm for only sputtered SnO_2_ sensor), SGO1b (23 nm for sputtered SnO_2_+1 bilayer PEI/GO sensor), SGO2b (32 nm for sputtered SnO_2_+2 bilayers PEI/GO sensor) and SGO4b (47 nm for sputtered SnO_2_+4 bilayers PEI/GO sensor) is presented in [116]. These data correspond to a shift increment of 35.29%, 88.24% and 176.47% in the studied range, respectively, which leads to an improvement of sensitivity thanks to the polymeric coatings obtained by LbL in comparison with an only sputtered coating. A summary of the optical setup as well as the resultant shift of the LMR band for the different optical fiber sensors is shown in Figure 15.

Other novel works that detect organic vapors by using the LMR technology as a function of the resultant shift of this absorption band when the device is exposed to distinct alcohols vapors can also be found in the literature [117,118]. In [117] the construction process of a LMR based optical sensor doped with a specific organometallic compound with the following chemical structure Au_2_Ag_2_(C_6_F_5_)_4_(C_6_H_5_C≡CC_6_H_5_)_2_]_n_ for the detection of three different VOCs (methanol, ethanol and isopropanol) is presented. The experimental results indicate that linear approximations were calculated for each type of alcohol, showing a specific sensitivity of 0.417 nm·ppm^−1^ (ethanol), 0.520 nm·ppm^−1^ (methanol) and 0.263 nm·ppm^−1^ (isopropanol), respectively, with a low hysteresis. Another different organometallic compound with the following chemical structure [Au_2_Ag_2_(C_6_F_5_)_4_(NH_3_)_2_]_n_ has been used in [118] for the detection of the same type of alcohols (methanol, ethanol and isopropanol), although the resultant sensitive coating for VOCs detection is different, as it can be appreciated in Table 2. 

Another interesting work for selective methanol gas detection by using a U-bent optical fiber modified with a silica nanoparticle multilayer is presented in [119]. In this work, tetrakis(4-sulfophenyl) porphine (TSPP) has been infused to a multilayer structure composed of PAH and SiO_2_ nanoparticles because the increase of the surface area by porous multilayer makes possible an enhancement of the diffusion of the target molecules into the film. The results corroborate that the resultant U-bent optical fiber showed very high selectivity against methanol among other alcohols widely used in chemistry laboratories. This same infusion mechanism by using TSPP is presented in [120], although in this work it is implemented for a different sensitive coating (PDDA/SiO_2_) which is coated onto a long-period grating (LPG) optical fiber, showing a high sensitivity to ammonia. It is important to remark that this specific analyte (ammonia) is one of the major metabolic compounds and a high-sensitivity for its detection has been emphasized recently due to its correlation with some specific diseases [121,122]. 

Other works are based on the use of TSPP as an anionic countpart during the LbL fabrication process [123,124,125] for gas sensing applications. In [123] is presented an optical fiber sensor to measure the gas emitted from human skin, being this approach of great interest for distinguishing the skin odors of different people as well as their altering physiological conditions after alcohol consumption. In [124] a porphyrin-nanoassembled fiber-optic sensor fabrication for ammonia gas detection is presented, showing a high selectivity of the sensor´s response to ammonia because there was no measurable changes when the sensor was exposed to other analytes such as toluene or pyridine. In [125] an optical fiber for high sensitivity ammonia detection is also presented, although by using other different optical configuration (U-shaped optical fiber sensor), showing a linear sensitivity (77.7 mV/ppm) to ammonia in the concentration ranges 1–100 ppm. 

**Figure 16:**
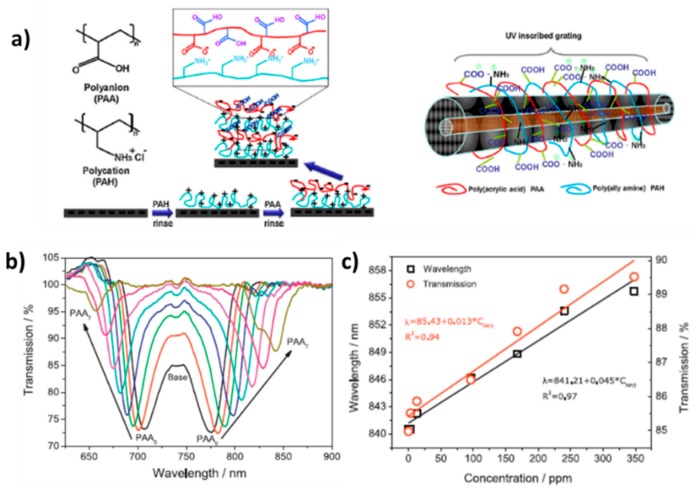
(**a**) Schematic illustration of film deposition using PAH and PAA polyelectrolytes onto an optical fiber LPG; (**b**) Changes of the TS optical fiber LPG during the deposition of PAA layers in the first and second resonance bands upon PAA deposition cycles; (**c**) Dependence of the wavelength shift of the resonance band and transmission spectrum (TS) changes of the film upon the ammonia gas concentration. Reprinted from [127] with permission.

According to ammonia detection, several works based on the combination of an LPG optical fiber and an adequate polyelectrolyte multilayer nanoassembled polyelectrolyte thin film can be also found in the literature [126,127,128]. In [126] a novel ammonia sensor composed of PDDA/PAA it is presented, where PAA can be used as a selective receptor for ammonia binding, which induces significant changes in the optical thickness, density of the film and electrostatic interactions. This same effect associated to PAA can be also appreciated in [127], where the ammonia binding is based on the acid-base interaction to free carboxylic acid groups of PAA, influencing in the transmission spectrum of the LPG. In this work PAH is used as the positive polyelectrolyte for ammonia detection instead of PDDA [126]. In Figure 16 a scheme of the deposition process onto the LPG optical fiber (Figure 16a), the changes of the transmission spectrum (TS) of optical fiber LPG during the fabrication of the polyelectrolyte alternate thin film (Figure 16b) and the resultant changes of the transmission spectrum of the TS of the LPG with the resultant relative wavelength shifts after exposition to variable ammonia concentrations (Figure 16c) can be appreciated.

In [128] a morphological study of both sensitive coatings [PDDA/PAA] and [PAH/PAA] is presented in order to clarify the changes of the optical transmission (OT) induced by ammonia gas adsorption. The main conclusion derived from this work is that the shrinkage of the PAA-anchored LbL films can be induced by the adsorption of amine analytes, becoming an important factor of the optical transmission increase (film density increase).

**Figure 17:**
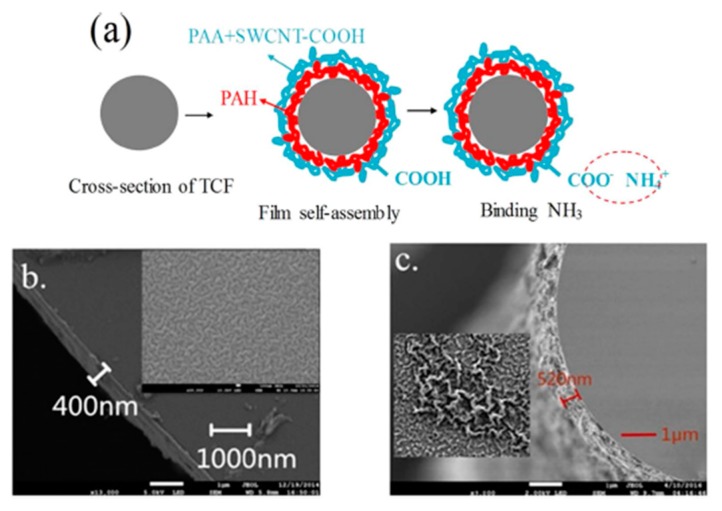
(**a**) Cross-sectional view of the TCF section; (**b**) The scanning electronic microscope (SEM) image of the cross-section of the fiber coated with (PAA/PAH)_10_ film. The inset shows the surface morphology; (**c**) SEM image of the cross-section of the fiber coated with (PAH/PAA)_2_ + [PAH/(PAA + SWCNTs-COOH)]_8_ film. The inset shows the surface morphology. Reprinted from [129] with permission.

**Figure 18:**
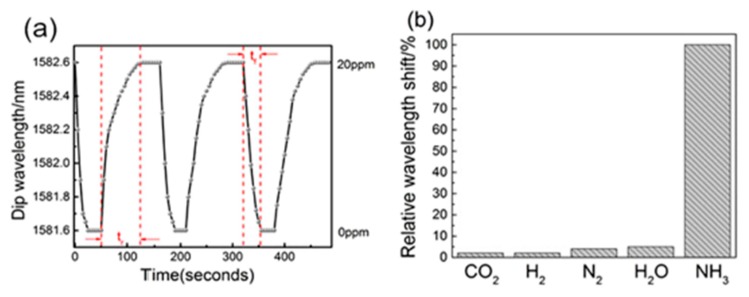
(**a**) Dynamic responses of the thin-core fiber interferometer ammonia sensor; (**b**) Relative wavelength shifts of the transmission spectrum of the TCF when is exposed to ammonia and other analytes (CO_2_, H_2_, N_2_ or H_2_O). Reprinted from [129] with permission.

Another work for ammonia gas sensing with high sensitivity can be found in [129]. In this article, a thin-core fiber modal interferometer has been made by fusion splicing a small section of thin-core fiber (TCF) between two standard single mode fibers (SMF). The outer surface of the TCF is coated by using the LbL technique and the resultant sensing film is composed of poly(acrylic acid) (PAA), poly(allylamine) (PAH) and single-walled carbon nanotubes(SWCNTs-COOH), as it can be appreciated in Figure 17. The dynamic response (excellent reversible and reproducible performance) as well as the selectivity (no remarkable changes to other components in the atmosphere such as CO_2_, H_2_, N_2_ or H_2_O) of the resultant sensor can be appreciated in Figure 18. 

A novel work for identification and quality assessment of beverages using a long period grating fiber-optic sensor modified with a mesoporous thin film is presented in [130]. The sensing film deposited onto LPG is composed of PAH and silica nanospheres (SiO_2_NPs). The presence of PAH in the sensing film imparts selectivity, whereas the use of SiO_2_NPs endow the film with high porosity and enhanced sensitivity. The experimental results corroborate that the LPG sensor is highly sensitive and selective for organic carboxylic acids found in grape-based beverages such as brandy, white and red wine, respectively. In this work, the sensing mechanism is based on the interaction between the organic carboxylic acids and the amine functional groups of the PAH polymer present in the PAH/SiO_2_ film on the LPG fiber. 

**Figure 19:**
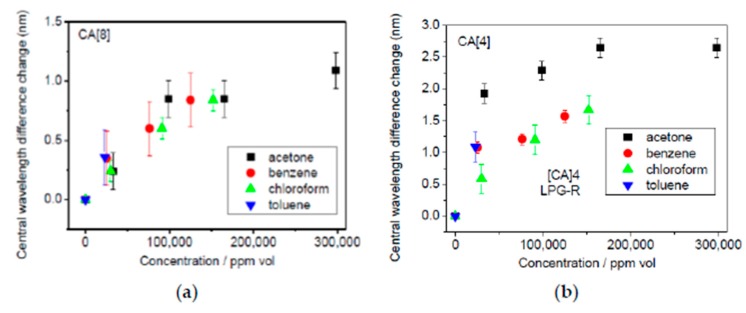
(**a**) CA[8] and (**b**) CA[4] Sensor’s response related to towards selected VOCs at different concentration levels (the error bars represent the standard deviation). Reprinted from [132] with permission.

Another interesting work for detection of specific volatile organic compounds (VOCs) such as chloroform and benzene vapors can be found in [131]. In this work, an optical fiber long period grating with a calixarene-anchored mesoporous thin film has been used for VOC detection. The mesoporous film consisted of an inorganic part (SiO_2_NPs) along with an organic moiety of PAH which has been infused with the functional compound *p*-sulphanatocalix[4]arene (CA[4]). The presence of CA[4] receptor into the LbL films allows sensitive measurements of VOCs with high reversibility and rapid response times. The sensing mechanism is based on the measurement of the refractive index change induced by the complexation of the VOCs with the CA[4] receptor. This same sensing mechanism has been used in [132], although in this work two different sizes of calixarene molecules such as CA[4] and CA[8], respectively, have been employed for the detection of individual VOCs and their mixtures evaporating from freshly painted surfaces. The experimental results indicate that although neither of the sensors can discriminate between individual VOCs, a different response has been observed to two different commercial paint products, suggesting that the sensors can distinguish between different mixtures. In addition, the CA[8] sensor exhibited a smaller response than CA[4], which is most likely a result of the lower amount of CA[8] infused into the PAH/SiO_2_ films. As it can be observed in Figure 19, the biggest wavelength shift has been observed for acetone, whereas the lowest wavelength shift has been observed for chloroform in the CA[4] sensor. In addition, no differences have been observed between individual VOCs for the CA[8] sensor. 

In order to have a better understanding of the different optical fiber sensors for gas and VOCs detection analyzed in this section, a summary of the different sensitive coatings employed as well as the optical structure and the sensing mechanism for the specific parameter detection can be clearly appreciated in Table 2. 

### 3.2. Biological and Biochemical Sensing Applications

One of the most essential biological materials is deoxyribonucleic acid (DNA) because it controls the heredity of life and the detection of its base sequence is of great importance in many different fields such as criminology, genetics, food safety or pathology, among others [133,134]. A novel label-free DNA sequence detection method on the surface of an optical fiber tip can be found in [135,136]. In these works, the principle of operation is based on the hybridization of a complementary DNA strand to immobilized DNA probes. According to this, firstly a single stranded capture DNA is immobilized into the multilayer structure of PAH/PSS (known as probe), and secondly, the optical fiber tip is immersed into the complementary DNA strand. Once the complementary DNA sample is hybridized, the resultant thickness of the probe is increased, so an adequate detection of the thickness changes indicates that the hybridization process has been successfully performed. The experimental results demonstrate sequence specificity and sensitivity, being possible to detect DNA quantities as small as 1.7 ng with short hybridization time [136]. Other interesting work can be found in [137] where it is the first time that an alternative DNA detection technique is presented by means of the use of label-free lossy mode resonance (LMR)-based optical fiber refractometers. The utilization of LMR-based optical fiber refractometers can be used as a good alternative for DNA detection by means of the immobilization of the adequate DNA probe because the refractive index is varied due to the presence of the complementary DNA, indicating that the hybridization process has been successfully performed. 

Other novel works are based on the development of optical fiber biosensors for the detection of immunoglobulins [138,139,140,141,142,143,144]. A representative example can be found in [138] where a biosensor for rapid detection of human immunoglobulin G (HIgG) is presented. In this work, the optical fiber biosensor has been constructed by sandwiching a section of a thin core optical fiber (TCSMF) between two single mode fibers (SMFs). The results indicate that the best sensitivity of HIgG detection was achieved for a wavelength shift of 16.92 nm/(mg/mL) and the corresponding limit of detection (LOD) was 0.59 mg/L, showing a great specificity because a negligible wavelength shift to NaCl solution or bovine serum albumin (BSA) has been observed.

**Figure 20:**
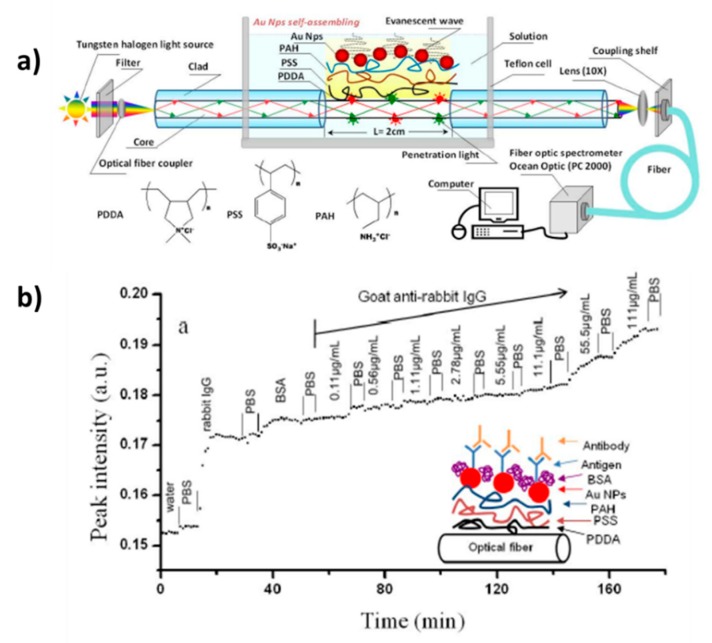
(**a**) Experimental setup of the optical fiber LSPR sensor; (**b**) LSPR biosensing of different concentrations of goat anti-rabbit IgG (0.11–100 µg/mL). Reprinted from [139] with permission.

In [139] an optical fiber LSPR biosensor prepared by gold nanoparticle assembly on polyelectrolyte multilayer which is used for the detection of goat anti-rabbit IgG is presented. In this work a trilayer polyelectrolyte structure composed of PDDA, PSS and PAH has been selected as a linker for the metallic gold nanoparticles (AuNPs) and these AuNPs are perfectly assembled in the polyelectrolyte structure with a well-defined Localized Surface Plasmon Resonance (LSPR) band in the visible region. A scheme of the experimental setup of the resultant optical LSPR sensor as well as the evolution in intensity of the LSPR band at different concentrations of goat anti-rabbit IgG can be appreciated in Figure 20. The experimental results indicate that the lowest detection concentration of goat anti-rabbit IgG was 11.1 ng/mL

A novel fiber-optic IgG biosensor based on the modulation of a LMR by the reaction between an IgG and its corresponding anti-IgG is proposed in [136], being the first time that a LMR has been used to study a biological reaction. The results indicate that there is a wavelength shift of 10 nm when detecting immunoglobulins with a response time of 12 min for a 50 µg/mL concentration. The results presented in [141,142] are based on the self-assembly of polymer/nanoparticles films for the fabrication of fiber-optic sensors to IgG detection. In these works, the nanofilm assembly as well as the antigen-antibody binding process has been studied using surface plasmon resonance (SPR) technique, and as a result an increase of the refractive index (measured as plasmon shift) has been appreciated which is indicative that there is an increasing thickness of the adsorbed films. In addition, the fiber optic SPR sensor has been tested to different concentrations of IgG which is ranging from 10^−11^ to 10^−4^ M for variable sizes of the resultant metallic nanoparticles (20 nm Ag, 10 nm Au and 20 nm Au, respectively) [142].

**Figure 21:**
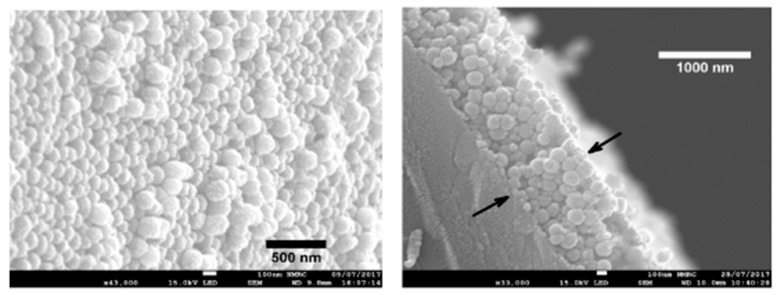
(**Left**) SEM of three layers of PAH/SiNPs with gold shell (scale bar = 500 nm); (**Right**) Cross-section of optical fibre with three layers of PAH/SiNPs with gold shell (scale bar = 1 μm). The arrows illustrate the thickness of the film, measured as 863 nm. Reprinted from [144] with permission.

References [143,144] are focused on the detection of human IgM (HIgM) by using optical fiber long period grating sensors. First of all, it has been scientifically corroborated that IgM has a very important clinical value in the diagnosis of diseases because is one of the earliest antibodies in appearing during the course of an infection. In addition, this specific immunoglobulin is mainly found in the lymph fluid and blood, being a very effective neutralizing agent in the early stages of disease [145]. A sensitive coating composed of PAH and silica core gold shell nanoparticles (SiO_2_@AuNPs) can be found in [143], and the resultant LPG biosensor (operating at the phase matching condition) showed detectable wavelength shifts for three different concentration of human IgM such as 19 nM, 83 nM and 0.3 µM, respectively. In [144] is also presented a LPG by using the same sensitive coating of PAH/SiO_2_@AuNPs (see Figure 21). 

**Figure 22:**
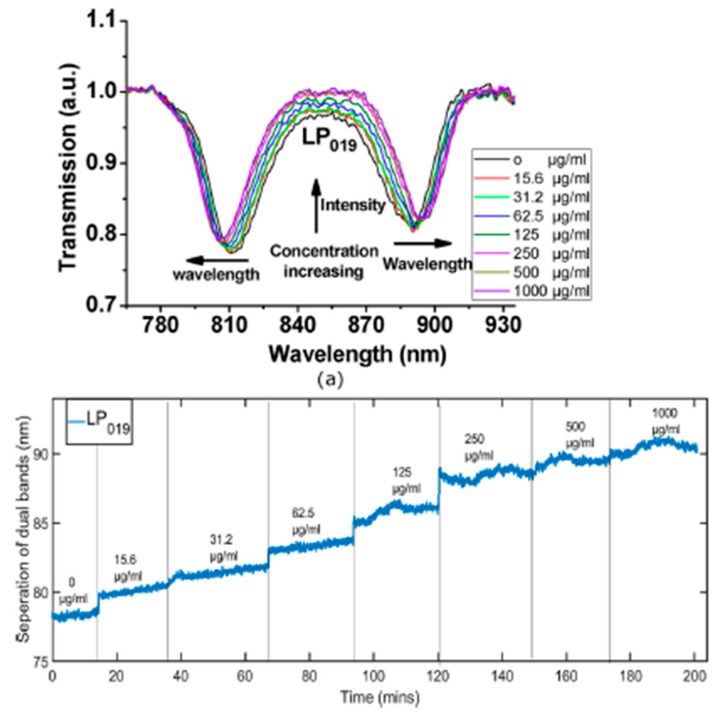
(**a**) Transmission spectrum of the LPG in different concentrations of IgM; (**b**) The separation of dual bands at LP_019_ in response to different concentrations of IgM suspension solution. Reprinted from [144] with permission.

In addition, the transmission spectrum of the LPG sensor at different concentrations of IgM can be appreciated in Figure 22 when the concentration was varied from 15.6 µg/mL (16.42 nM) up to 1 mg/mL (1 µM), respectively. The experimental results indicated that the central wavelength of P_019_ at 850 nm showed an increase in transmission value and the separation of the dual bands at LP_019_ was wider as increasing the concentration of IgM. The LPG sensor exhibits a sensitivity of 11 nm/(ng/mm^2^) with a detection limit of 15 pg/mm^2^. In this work [144] the same sensitive coating for the detection of streptavidin, once the biotin is covalently bonded to the surface of the gold shell, was also used. The LPG sensor showed a sensitivity of 3.88 nm/(ng/mm^2^) and a detection limit of 0.86 pg/mm^2^, being this limit of detection 22 times lower than the one reported in [146] (a limit of detection of 19 pg/mm^2^) by using the same optical configuration (LPG), although with a different the functionalization chemical route of the LPG with silica core gold shell nanoparticles. Other LPG configuration for streptavidin detection can be found in [147] where a multilayer coating composed of PAH and poly[1-[4-(3-carboxy-4-hydroxyphenylazo) benzenesulfoamido]-1,2-ethanediyl, sodium salt (PCBS) has been use as sensing layer. 

**Figure 23:**
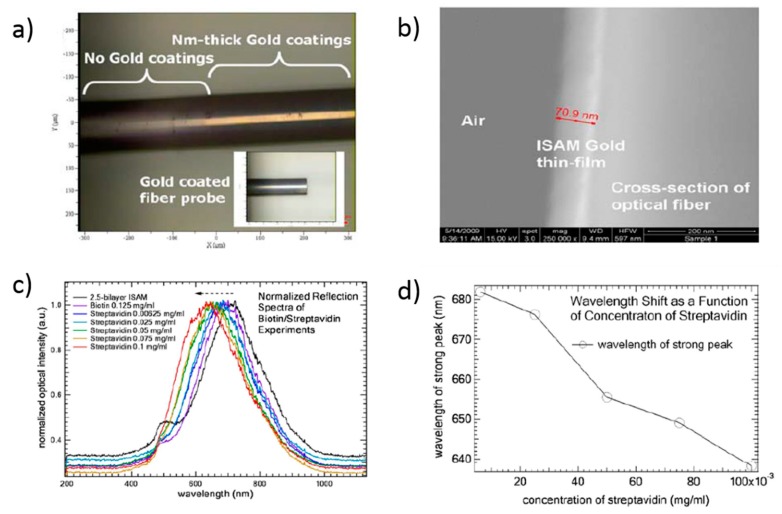
(**a**) Color picture of ISAM gold film coated fiber probe imaged by bright field microscopy, inset is a cleaved fiber probe with gold coatings; (**b**) SEM picture of cross-section of a cleaved optical fiber coated by 10-bilayer ISAM gold films; (**c**) Normalized reflection spectra measured in air as a function of the concentration of streptavidin solution using the same fiber probe. Black dash arrow indicates the direction of the wavelength shift of the longer-wavelength strong peak as concentration of streptavidin solution increases; (**d**) Wavelength shift as a function of concentration of streptavidin. Reprinted from [148] with permission.

Other work based on LSPR biosensor for streptavidin detection is presented in [148] where the sensitive coating is composed of [PAH/AuNPs] and it is deposited onto the end-face of a multimode optical fiber using reflection mode. The experimental results indicate that the wavelength of the strong LSPR peak red-shifts to shorter wavelengths as the concentration of streptavidin is increased, as it can be seen in Figure 23. 

In [149] a novel highly sensitive protein sensor on a tapered optical fiber modified with a Au-based nanocoating is presented. In this work it is remarked that the LbL coating morphology with the AuNPs provides a higher specific surface area for the adhesion of more biotin molecules which is a key factor for a further streptavidin (SV) absorption capacity in these LbL coatings. The biosensor showed a high sensitivity to SV with the lowest measured concentration levels below 2.5 nM with a limit of detection of 271 pM. 

Other well-known specific biomolecular recognition is produced between concavidin A (Con A) and ribonuclease B (RNase B), and in [150] a gold nanosphere-based fiber optic LSPR probe for its qualitative and quantitative detection in real real-time is presented. In this work, gold nanoparticles are immobilized into a polymeric matrix and then, the sensing surface is further functionalized with RNase B. The experimental results indicate that there is decrease in intensity of the LSPR band when it is added Con A, which is consistent that Con A is bound specifically to RNase B on the sensing surface. 

**Figure 24:**
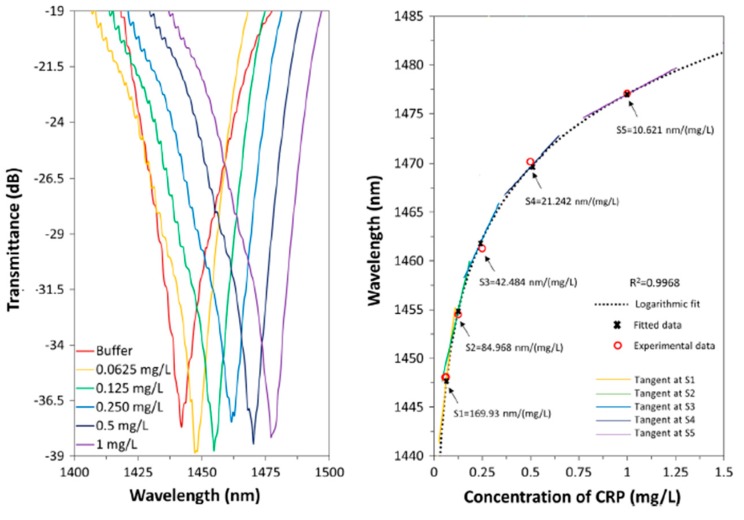
Spectral response of the sensor when the sensitive region is exposed to different concentration of CRP. Reprinted from [153] with permission.

In addition, optical fiber biosensors for the detection of specific parameters such as glucose [151], anti-gliadin antibodies [152] or C-reactive protein [153,154] can be also found. Their detection is of great interest because these parameters are directly associated to diseases such as diabetes, celiac disease, cardiovascular disease or coronary heart disease risk, among others. In [151] a fiber glucose biosensor which is sensible over a range of glucose concentrations between 0.06 and 2 mM is presented, being the sensitive coating composed of PAH combined with Prussian Blue (PB) as polycationic solution and enzyme glucose oxidase (GOx) as polyanion solution, respectively. In [152] an optical fiber for detection of anti-gliadin antibodies (AGAs), in order to early diagnose of celiac disease by using LMR technology is presented. In this work, it was corroborated that the LMR shape depends on the geometry of the optical structure and its attenuation has been controlled by tuning the length of the device, being the best performance obtained tapered single-mode optical fibers. The results indicate that AGA concentrations of 5 ppm have been successfully detected, which represent lower values than those obtained with current celiac disease detection techniques. Finally, in [153] a high sensitive and selective C-reactive protein (CRP) detection by means of LMR based optical fiber devices is presented. In these works, the receptor layer has been fabricated using the LbL technique, consisted of a structure of PAH and PSS, followed by the CRP-aptamer layer for the further recognition of CRP. In Figure 24 it can be clearly appreciated that the resonance wavelength shifts towards higher wavelengths as the concentration of CRP is increased from 0.0625 mg/L up to 1 mg/L, respectively. In addition, from the response approximation equation obtained in this figure, it can be also calculated the sensitivity of the device in the vicinity of the studied concentrations, being the sensitivities of 69.93, 84.968, 42.484, 21.242 and 10.621 nm/(mg/L) for 0.0625, 0.125, 0.25, 0.5 and 1 mg/L, respectively. 

**Figure 25:**
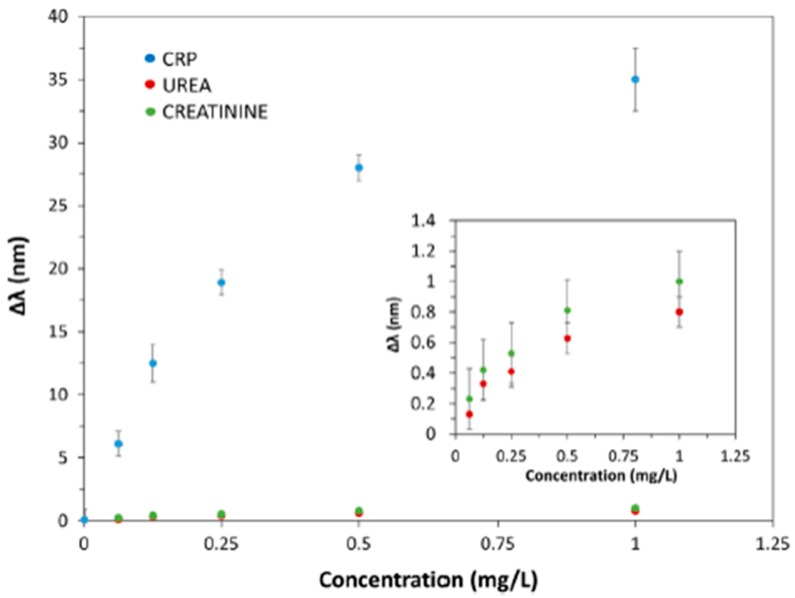
The shift in wavelength when the sensitive region is exposed of different concentration of CRP, Urea and Creatinine. Reprinted from [153] with permission.

Additionally, in order to study the selectivity of the sensor, it has been subjected to different solutions in presence of other molecules, such as urea and creatinine at the same concentrations used with CRP. In Figure 25 the LMR wavelength shift values for different concentrations of CRP, urea and creatinine are shown. The shift in resonance wavelength as a function of urea and creatinine solutions in each case are shown in the inset of the figure along with corresponding error bar at each point. An important consideration is that the values of the LMR shifts are less than 1 nm for urea and creatinine, which confirms that the sensitive layer (CRP-aptamer) is highly selective to CRP. These experimental results corroborate that the biosensor shows a high specificity to CRP and negligible interactions with other molecules (urea or creatinine), showing fast response time (61 s), good repeatability and reusability between different days. 

In order to have a better understanding of the different optical fiber sensors for biological and biochemical detection analyzed in this section, a summary of the different sensitive coatings employed as well as the optical structure and the sensing mechanism for the specific parameter detection can be clearly appreciated in Table 3. 

## 4. Conclusions 

In this work an overview of the implementation of LbL coatings for the design and development of novel optical fiber devices for the detection of chemical, biological and biochemical parameters is presented. In this regard, the use of optical fiber technology provides a powerful alternative sensing platform due to the inherent potential properties such as easy multiplexing, light weight, small size, immunity to electromagnetic interferences and long-distance transmission, among others. Different optical fiber configurations (mostly reflection, transmission, taper, U-bend, LPGs) are used for the deposition of the sensitive coating by using the LbL technique. It is important to remark that this nanofabrication technique provides a good thickness control and a high versatility due to the possibility of incorporating a wide variety of chemical (metal, metal oxide, luminescent materials, dyes) or biochemical substances (proteins, aptamers, antibodies, antigens) into the corresponding multilayer structure which can optimize the characteristics of the sensing films such as sensitivity, selectivity, response time or even the range of measurement. This review demonstrates that the LbL technique is a powerful tool to create functional coatings for optical fiber sensors, with enhanced properties respect to the bulk materials, making possible the achievement of highly sensitive optical devices for pH measurement, the identification of gases and volatile organic compounds (VOCs) or even for highly specific biological applications.

## Figures and Tables

**Table 1:** Summary of the different optical fiber pH sensors with their corresponding sensitive coating, optical structure, sensing mechanism and pH range of study.

Sensitive Coating	Optical Structure	Sensing Mechanism	pH Range	Reference
[PAH/PAA]	Reflection	Absorbance	3–9	[76]
[PAH/PAA]	Transmission	LMR	3–6	[81]
[PAH/PAA]	Taper	LMR	4–6	[84]
[PAH/PAA]	D-shape	LMR	4–5	[86]
[PAH/PAA]	D-shape	LMR	6–7	[86]
[PAH/PAA-AuNPs]	Transmission	LSPR and LMR	4–6	[88]
[PAH+PB/PAA]	LPGs	Wavelength shift	4–7	[91]
[PAH + NR/PAA]	U-bend	Absorbance	5–10	[93]
[NR/PAA]	Reflection	Absorbance	3–9	[76]
[NR/PAA]	Reflection	Wavelength shift	5.5–7.5	[94]
[PAH/BY]	Reflection	Wavelength shift	6.80–9	[95]
[PAH/BY]	U-bend and straight	Wavelength shift	7–9	[96]
[PAH/PAA+HPTS]	Taper	Fluorescence	3–7	[75]
[PAH/SiO_2_] + [PAH/PAA+HPTS]	Taper	Fluorescence	3–7	[75]
[PAH+DABCO/PAA+HPTS+DABCO]	Taper	Fluorescence	3–7	[97]
[PDDA/PAA]	TFBG	Absorbance	4.66–6.02	[98]
[PDDA/PAA]	MZ	Wavelength shift	4.48–5.66	[99]
[PVPMC+PDDA/PAA]	MZ	Wavelength shift	4.48–5.66	[99]
[PDDA/PSS + PDDA/PEC^−^]	MZ	Wavelength shift	2–11	[100]
[PEI/SA]	MZ	Wavelength shift	2–11	[101]

**Table 2:** Summary of the different optical fiber sensors to detect VOCs and gases with their corresponding sensitive coating, optical structure, sensing mechanism and parameter of study.

Sensitive Coating	Optical Structure	Sensing Mechanism	Parameter Detection	Reference
[PAH + porous glass beads/PAA]	Reflection	Fluorescence	Oxygen	[108]
[PAH/SDS-Pt-TFPP]	Reflection	Fluorescence	Oxygen	[109]
[PAH/SDS-Pt-TFPP]	Reflection	Fluorescence	Oxygen	[110]
[PDDA/SDS-Pt-TFPP] [PEI/SDS-Pt-TFPP] [PAH/SDS-Pt-TFPP]	Reflection	Fluorescence	Oxygen	[111]
[P(+)/SDS-Pt-TFPP] [P(+)/SDS-Pt-TFPP/(P(+)/PAA)_N_]_4_+[P(+)/SDS-Pt-TFPP] N = 1, 2, 3 and P(+) = PAH, PDDA, PEI	Reflection	Fluorescence	Oxygen	[112]
[PEI/GO]	Transmission	LMR	Ethanol	[115]
[PEI/GO]	Transmission	LMR	Ethanol	[116]
[PAH/[Au_2_Ag_2_(C_6_F_5_)_4_(C_6_H_5_C≡CC_6_H_5_)_2_]_n_	Transmission	LMR	Methanol Ethanol Isopropanol	[117]
[PAH/PAA/[Au_2_Ag_2_(C_6_F_5_)_4_(NH_3_)_2_]_n_/PAA	Transmission	LMR	Methanol Ethanol Isopropanol	[118]
TSPP-infused PAH/SiO_2_	U-bend	Intensity	Methanol	[119]
TSPP-infused PDDA/SiO_2_	LPG	Intensity	Ammonia	[120]
[PAH/TSPP]	Transmission	Intensity	Skin emanation	[123]
[PDDA/TSPP]	Transmission	Intensity	Ammonia	[124]
[PDDA/TSPP]	U-bend	Intensity	Ammonia	[125]
[PDDA/PAA]	LPG	Wavelength shift	Ammonia	[126]
[PAH/PAA]	LPG	Wavelength shift	Ammonia	[127]
[PAH/PAA] [PDDA/PAA]	LPG	Wavelength shift	Ammonia	[128]
[PAH/PAA]+[PAH/(PAA+SWCNTsCOOH)]	MZ	Wavelength shift	Ammonia	[129]
[PAH/SiO_2_]	LPG	Wavelength shift	Carboxylic acids in beverages	[130]
Calix[4]arene-infused PAH/SiO_2_	LPG	Intensity	Chloroform Benzene	[131]
Calix[4]arene-infused PAH/SiO_2_ Calix[8]arene-infused PAH/SiO_2_	LPG	Wavelength shift	Chloroform Benzene Toluene Acetone	[132]

**Table 3:** Summary of the different optical fiber sensors to detect biological and biochemical parameters with their corresponding sensitive coating, optical structure, sensing mechanism and parameter of study.

Sensitive Coating	Optical Structure	Sensing Mechanism	Parameter Detection	Reference
[PAH/PSS]	Transmission	Fiber cavity thickness	DNA	[135]
[PAH/PSS]	Transmission	Fiber cavity thickness	DNA	[136]
[PAH/PSS]	Transmission	LMR	DNA	[137]
[PDDA/PSS]	MZ	Wavelength shift	HIgG	[138]
[PDDA/PSS/PAH/AuNPs]	Transmission	LSPR intensity	Goat anti-rabbit IgG	[139]
[PAH/PSS]	Reflection	LMR	IgG	[140]
[PAH/PSS] + [PAH/AuNPs]	Reflection	Refractive index changes	IgG	[141]
[PAH/PSS] + [PAH/AgNPs or AuNPs (variable size of 10 and 20 nm)]	Reflection	Wavelength shift	IgG	[142]
[PAH/SiO_2_@AuNPs]	LPG	Wavelength shift	HIgM	[143]
[PAH/SiO_2_@AuNPs]	LPG	Wavelength separation	HIgM	[144]
[PAH/SiO_2_@AuNPs]	LPG	Wavelength shift	Streptavidin	[144]
[PAH/SiO_2_@AuNPs]	LPG	Wavelength shift	Streptavidin	[146]
[PAH/PCBS]	LPG	Wavelength shift	Streptavidin	[147]
[PAH/AuNPs]	Reflection	Wavelength shift	Streptavidin	[148]
[PAH/SiO_2_NPs] + [AuNPs/PSS]	Taper	Wavelength shift	Streptavidin	[149]
[PDDA/PSS/PAH] + AuNPs	Transmission	LSPR intensity	Con A	[150]
[PAH+PB/GOx]	Reflection	Fabry-Perot cavity	Glucose	[151]
[PAH/PAA] + [PAH/PSS]	Taper	LMR	Anti-gliadin antibodies	[152]
[PAH/PSS]	D-shaped	LMR	C-reactive protein	[153]
